# Vertical Silicon Nanowire Field Effect Transistors with Nanoscale Gate-All-Around

**DOI:** 10.1186/s11671-016-1396-7

**Published:** 2016-04-19

**Authors:** Youssouf Guerfi, Guilhem Larrieu

**Affiliations:** LAAS-CNRS, Université de Toulouse, CNRS, 7 av. Roche, Toulouse, 31077 France

## Abstract

Nanowires are considered building blocks for the ultimate scaling of MOS transistors, capable of pushing devices until the most extreme boundaries of miniaturization thanks to their physical and geometrical properties. In particular, nanowires’ suitability for forming a gate-all-around (GAA) configuration confers to the device an optimum electrostatic control of the gate over the conduction channel and then a better immunity against the short channel effects (SCE). In this letter, a large-scale process of GAA vertical silicon nanowire (VNW) MOSFETs is presented. A top-down approach is adopted for the realization of VNWs with an optimum reproducibility followed by thin layer engineering at nanoscale. Good overall electrical performances were obtained, with excellent electrostatic behavior (a subthreshold slope (SS) of 95 mV/dec and a drain induced barrier lowering (DIBL) of 25 mV/V) for a 15-nm gate length. Finally, a first demonstration of dual integration of n-type and p-type VNW transistors for the realization of CMOS inverter is proposed.

## Background

The continuous demand of high-performance and low-power devices necessitates integration density enhancement, which pushes the CMOS technology to the ultimate nanoscale size dimension. Nanowire (NW) MOSFETs [1–5] are considered the most promising candidates to pursue the downscaling of MOS transistors, outperforming of triple gate FinFET architectures for sub-7-nm technology node. Indeed, nanowire architecture is more suitable for gate-all-around configuration to preserve the device immunity against the short channel effects (SCE) at such scaled dimensions [6, 7]. This architecture is speculated to bring CMOS scaling to the end of the transistor roadmap [8]. The NW manufacturing method could be divided into two main approaches: bottom-up (BU) and top-down (TD). The first one suffers from process complexity [9] and metallic contamination issues since metallic nanoparticles are used as a catalyst. In contrast, the TD approach, using conventional microfabrication tools, is more suitable for nanoelectronic applications. It offers perfect control over dimensions, localization, and orientation which leads to highly efficient NW MOSFET devices [10–12]. The integration of the NW-based MOSFETs can be horizontal or vertical. From design analysis, vertical integration could give a better integration density of 50 % over the horizontal one [13, 14]. Moreover, gate-all-around definition in vertical configuration, even at nanoscale, is not defined by high-resolution lithography but simply by the thickness of deposited gate material [15, 16]. In this letter, we present a large-scale process for manufacturing of GAA vertical silicon nanowire (VNW) MOSFETs with a 15-nm gate length. The electrical performances will be discussed, in particular the influence of nanowire diameter on the device operation. Finally, a proof of concept for a CMOS inverter will be presented.

## Methods

**Fig. 1:**
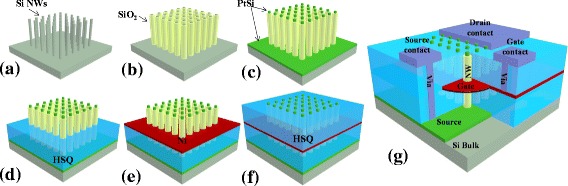
Schematic 3D illustrations of the process steps of Si VNW GAA MOSFET. **a** Si VNW network patterning. **b** Gate oxide definition. **c** Metallic S/D contacts. **d** Gate to source isolating spacer. **e** Gate-all-around definition. **f** Drain to gate isolating spacer. **g** Metallic vias and contact pads

In Fig. 1, the fabrication steps of a Si VNW MOSFET are presented. After the realization of the VNWs (Fig. 1a), an SiO_2_ layer acting as gate oxide is grown and remained only on the sidewalls of the nanowires forming a Si–SiO_2_ core–shell structure (Fig. 1b). Then, the realization of silicided contacts at both sides of the Si VNWs for the realization of low resistive source/drain contacts is shown (Fig. 1c). The next step consists of the realization of the source to gate insulating spacer (Fig. 1d), followed by the formation of the metallic gate-all-around (Fig. 1e) and by the gate-drain insulating spacer (Fig. 1f). Finally, the electrical contacts are defined on top of the architecture and are connected to the bottom and gate contact extensions using vias through the insulation layers (Fig. 1g). A conventional photolithography process has been developed to enable large-scale manufacturing of Si VNW MOSFETs.

**Fig. 2:**
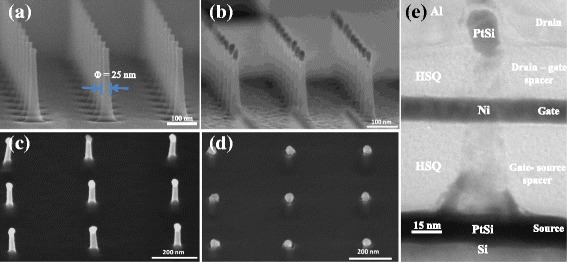
Tilted SEM images of **a** Si VNW network of 25-nm diameter, **b** PtSi contacts at both side of VNW (**c**) and (**d**), respectively, gate to source and drain to gate insulating spacer formed by HSQ planarization technique and **e** TEM cross-section image of multilayer stacks implemented on VNW arrays

The starting substrate is a 4-in. Si (100) wafer, boron doped at 2 × 10^19^ atm/cm^3^, to realize p-type MOSFETs. The Si VNWs are realized based on a top-down approach, through the transfer of hard mask by plasma etching onto the silicon substrate. First, electron beam lithography is used to pattern a high-resolution inorganic resist, hydrogen silsesquioxane (HSQ), in order to fabricate tiny nanopillars with high aspect ratio, up to 7.5, in 150-nm HSQ thickness. A perfectly circular shape with a dispersion of 1.62 nm (1σ) was obtained thanks to an original design—namely a star-like design—which consists of lines starting and finishing in the center of the pattern to force the symmetry of the nanopillars and promote an homogeneous distribution of Gaussian energy [17]. The electron beam exposure is carried out with a Raith 150 system at low energy (20 kV) with a base dose of 300 μC/cm^2^ and a current of 120 pA. The revelation of the resist after exposure is performed in tetra methyl hydroxyl ammonium solution at high concentration (25 % in water) to enhance the pattern contrast. The Si VNWs are obtained by performing plasma etching using chlorine chemistry, low pressure (2 mTorr), and 80 W bias power in pure capacitive coupling plasma (CCP) to promote the anisotropy of nanowire sidewalls [18]. VNWs with 94 % of anisotropy are obtained without any visible damage or rugosity on the surface of the NWs. The silicon etching rate is 80 nm/min with a selectivity Si:HSQ = 2:1. The residual resist is stripped in diluted hydrofluoric acid solution. Finally, VNWs are thinned by a sacrificial wet oxidation at 850 °C for 10 min. Thanks to the stress-retarded oxidation effect during the oxide growth [19], the nanowire thinning is controllable [20] and the new interface is free from defects generated by the plasma step. Nanowire networks with maximal yield and reproducibility, without surface roughness or geometrical irregularities, are obtained. In Fig. 2a, a Si VNW network with a 25-nm diameter is presented with an excellent reproducibility. The gate oxide is realized by dry oxidation at 725 °C over 30 min. The thickness of the grown SiO_2_ is around 4 nm and is wrapping the entire VNW’s structure.

Before the implementation of platinum silicides (PtSi) for the realization of metallic source/drain contacts, the SiO_2_ layer is etched selectively by CCP mode plasma at both sides of the VNWs using a fluorine chemistry (CHF3:CF4:Ar = 20:20:10). The source contact is defined by a lift-off process. A 10-nm platinum layer is deposited by electron beam evaporation (anisotropic deposition), followed by the silicide reaction activation using a rapid thermal annealing (RTA) at 500 °C during 3 min under N_2_H_2_ (96 % 4 %) atmosphere. The VNW sidewalls are cleaned from platinum contaminations by a selective wet etching in aqua regia solution. Figure 2b presents a VNW network with silicided contacts at both sides of the VNWs with clean sidewalls. The 3D process completion must go through a perfect mastery of nanoscale layer engineering. To this end, an innovative planarization method to realize the source to gate isolation spacer was developed using the HSQ as a dielectric material [21]. It is characterized by a low viscosity, providing excellent filling properties. Moreover, the HSQ is a CMOS-compatible material that offers a low dielectric constant (*k* ≈ 2.7), minimizing the parasitic capacitances. The planarization process was performed by chemical etching in highly diluted hydrofluoric acid (HF) in deionized water (1:1000). This approach has been preferred to plasma etching or chemical mechanical polishing (CMP); both approaches are widely used to planarize horizontal architectures but are not adapted to 3D architectures. However, the etching of the HSQ in diluted HF needs to take over bubble encroachments on the dielectric surface issued from the releasing of the hydrogen gas during SiO_x_H_y_ etching that induces severe degradations leading to a rough surface. To prevent the bubble encroachment, a cationic surface agent, benzalkonium chloride, has been added to the diluted HF solution. This leads to the extraction of the gas bubbles as they appeared on the surface which then vanish in the solution. The resulting planarization is presented in Fig. 2c with a highly planarized HSQ layer over VNWs. It demonstrated an extremely flat layer, minimal roughness below 2 nm (measured by atomic force microscopy), without any degradation of the HSQ surface or of the VNWs. The etching rate of the HSQ is 2.3 nm/s, resulting in a highly controllable process. A 15-nm nickel layer is then deposited by electron beam evaporation over the gate to source spacer. The gate contact is performed by each back process after the definition of hard mask by photolithography step. The unprotected Ni layer is etched in chemical solution based on H_2_SO_4_:H_2_O_2_:EDI = 25:1:50 ml, followed by the photoresist stripping. For the proposed architecture, it is worth noting that the gate length is simply defined by the gate material thickness, without any high-resolution lithography step. A second spacer is then performed to insulate the gate to the drain contacts (Fig. 2d). Via openings are created by plasma etching in the dielectric to contact the metallic source and gate extensions. Finally, a 400-nm thick Al layer is deposited by sputtering followed by a chemical etch back in a solution of H_3_PO_4_:HNO_3_:EDI = 5:40:7. Finally, a forming gas anneal (FGA) (N_2_H_2_, 96 % 4 %) at 250 °C for 4 min is performed in order to passivate defects at both Si-SiO_2_ interface and at Si-PtSi contact interface [22].

## Results and discussions

A cross-section image of the device performed by transmission electron microscopy is presented in Fig. 2e and shows the 3D stacking composed of three conductive layers (source, gate, and drain contacts), separated by two insulating layers with homogenous thicknesses and without any defect or damage in the dielectric layers or wave effect at the vicinity of the NWs.

**Fig. 3:**
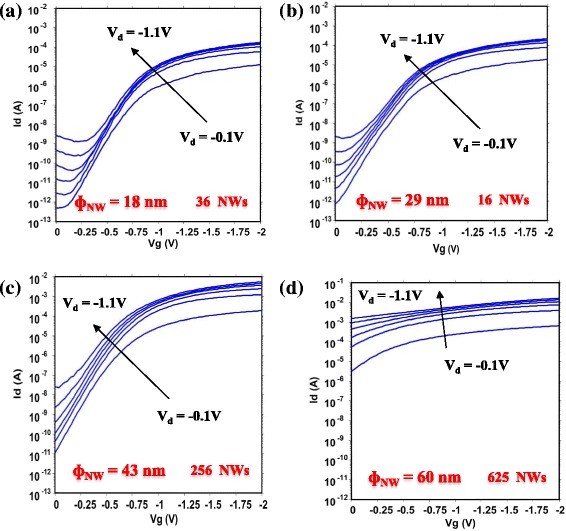
Transfer characteristics of GAA MOSFET on Si VNW with a diameter of **a** 18 nm, **b** 29 nm, **c** 43 nm, and **d** 60 nm

**Fig. 4:**
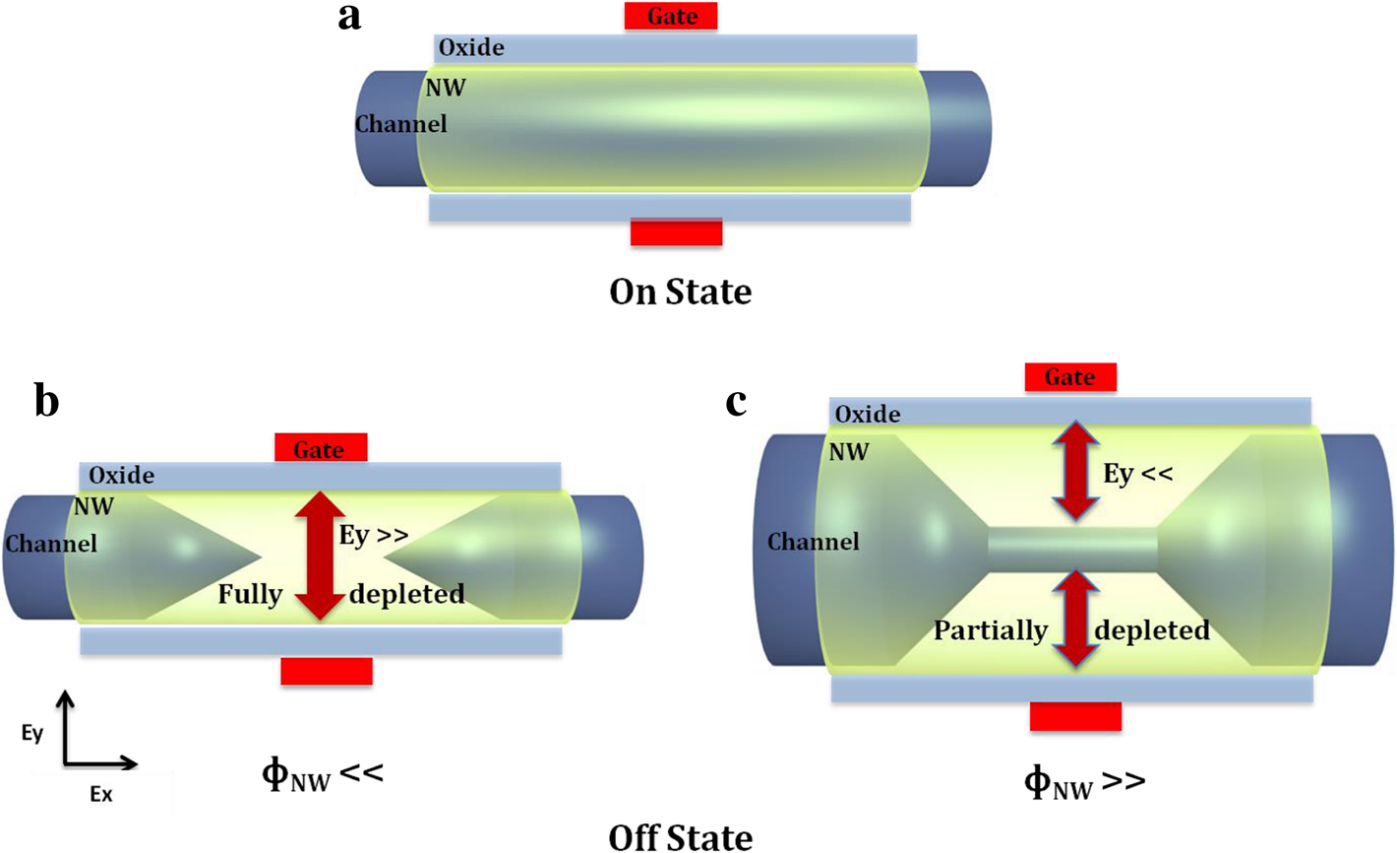
Schematic representation of the carrier density in the channel at **a** on-state regime and **b**, **c** off-state regime for thin and large NW diameters, respectively

Electrical characterizations of such 3D NW architectures with a nominal gate length of 15 nm have been performed with a Cascade prober and parametric analyzer Agilent 4156C. Static characteristics of devices with different NW diameters (from 18 to 60 nm) are given in Fig. 3. The p-type devices exhibit normally off behavior with high control over short channel effects with almost-ideal subthreshold characteristics (SS = 95 mV/dec and DIBL = 25 mV/V) for a device with NW diameter of 18 nm (Fig. 3a). The same characteristics are obtained when the NWs have a diameter of 29 nm (Fig. 3b) and 43 nm (Fig. 3c). The devices exhibit high drive current (normalized by the number and the diameter of NWs) of 431/472 μA/μm when the NW diameters are of 29 nm/43 nm, respectively. When the NW diameter becomes larger than 40 nm (Fig. 3d), the electrostatic control of the gate degrades quickly, and it is no longer able to turn off the device. Figure 4 presents a schematic representation of the carrier density in such a GAA NW configuration in order to explain the impact of the NW diameter on the transistor operation. The device operates in junctionless mode [23] (accumulation mode). When the transistor is turned on (Fig. 4a), the channel conduction is made by the entire section of the NW, which means that the larger the NW diameter is, the larger the drive current per NW is. When the device is turned off, a transverse electrical field (Ey), due to the electrostatic coupling between the gate (metal work function) and the potential in the channel region, induces the depletion of the channel. For narrow diameters (Fig. 4b), the field developed by the gate can deplete the entire volume of the NW. Nevertheless, when the NW is too large (Fig. 4c), the channel is only partially depleted by the gate. A conductive path from the source to the drain is remaining and the transistor cannot be turned off.

**Fig. 5:**
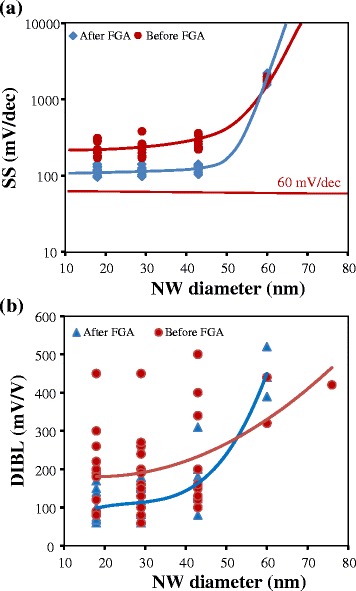
Impact of forming gas annealing on **a** SS and **b** DIBL as a function of NW diameters

**Fig. 6:**
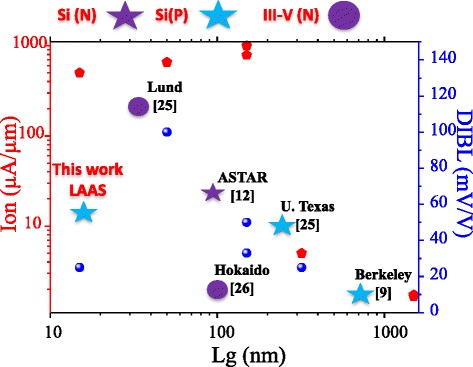
DIBL and ion state of the art of vertical nanowire transistors as a function of the gate length

Figure 5 summarizes the evolution of the SS (Fig. 5a) and DIBL (Fig. 5b) as a function of NW diameter, before and after the FGA. The defect passivation not only improves the immunity against short channel effects but also greatly reduces device to device variability. Low-frequency noise analysis [24] confirmed the absence of active oxide traps for most wires after the passivation step. Before FGA, the characteristics suffered high variability from device to device, which is supposed to come from the random distribution of interface defects in such miniaturized devices. A comparison to the state of the art (Fig. 6) of vertical nanowires MOSFET architecture [25–27] frames this result in favorable terms. Even if it is the most scaled demonstration published up to now, the immunity against short channel effect remains robust. Normalized drive current is also high when compared to p-type transistors. The vertical integration of nanowire transistors is not at the same level of technological maturity as planar/horizontal integration; however, this experimental demonstration shows that this 3D NW architecture should be considered a potential candidate for a sub-5-nm technology node [14].

**Fig. 7:**
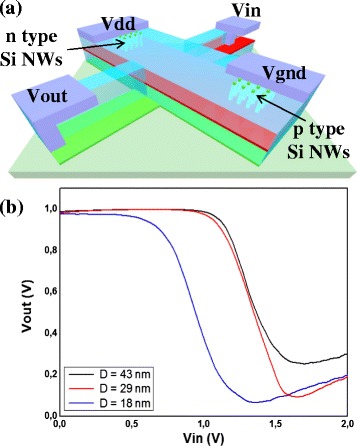
**a** Schematic 3D illustration of Si VNW CMOS inverter. **b** Transfer characteristics of inverters for different NW diameters

Finally, in the last section of this report, a first proof of concept for a CMOS inverter based on 15-nm GAA vertical transistors is presented in Fig. 7. Highly doped wells (2 × 10^19^ atm/cm^3^) with phosphorous and boron species are performed by conventional ion implantation in order to pattern n- and p-type Si VNWs on the same wafer. A similar transistor process, as described in Fig. 1, is implemented to realize CMOS inverters. Such a 3D architecture is schematically shown in Fig. 7a. The transfer characteristics of inverters implemented on different NW diameters are presented in Fig. 7b, showing regular inverter behavior with sharp commutation from high to low state. Contact engineering for NMOS counterpart is mandatory to enhance the switching characteristics of the inverter by implementing, for example, dopant segregation techniques [28] to reduce the large Schottky barrier to electrons of Pt silicide.

## Conclusions

In summary, a large-scale process of Si VNW MOSFETs with nanoscale GAA is presented. The nanowire arrays were made by a top-down approach, and the vertical transistor was realized by a successive engineering of nanoscale thin films using conventional UV lithography. The electrical performances demonstrated excellent electrostatic behavior of the device for a 15-nm gate length, with very good immunity against short channel effects. Finally, a proof of concept for a CMOS inverter by dual integration of n- and p-type Si VNWs was proposed.
